# BOLD Response Selective to Flow-Motion in Very Young Infants

**DOI:** 10.1371/journal.pbio.1002260

**Published:** 2015-09-29

**Authors:** Laura Biagi, Sofia Allegra Crespi, Michela Tosetti, Maria Concetta Morrone

**Affiliations:** 1 IRCCS Stella Maris Foundation, Calambrone, Pisa, Italy; 2 Department of Psychology, Vita-Salute San Raffaele University, Milan, Italy; 3 CERMAC and Neuroradiology Unit, San Raffaele Hospital, Milan, Italy; 4 Department of Translational Research on New Technologies in Medicine and Surgery, University of Pisa, Pisa, Italy; University of Oregon, UNITED STATES

## Abstract

In adults, motion perception is mediated by an extensive network of occipital, parietal, temporal, and insular cortical areas. Little is known about the neural substrate of visual motion in infants, although behavioural studies suggest that motion perception is rudimentary at birth and matures steadily over the first few years. Here, by measuring Blood Oxygenated Level Dependent (BOLD) responses to flow versus random-motion stimuli, we demonstrate that the major cortical areas serving motion processing in adults are operative by 7 wk of age. Resting-state correlations demonstrate adult-like functional connectivity between the motion-selective associative areas, but not between primary cortex and temporo-occipital and posterior-insular cortices. Taken together, the results suggest that the development of motion perception may be limited by slow maturation of the subcortical input and of the cortico-cortical connections. In addition they support the existence of independent input to primary (V1) and temporo-occipital (V5/MT+) cortices very early in life.

## Abstract 2

Although 7-wk-old infants do not perceive motion with fine sensitivity, this study shows that their brains have a well-established network of associative cortical areas selective to visual flow-motion.

## Author Summary

While it is known that the visual brain is immature at birth, there is little firm information about the developmental timeline of the visual system in humans. Despite this, it is commonly assumed that the cortex matures slowly, with primary visual areas developing first, followed by higher associative regions. Here we use fMRI in very young infants to show that this isn’t the case. Adults are highly sensitive to moving objects, and to the spurious flow projected on their retinas while they move in the environment. Flow perception is mediated by an extensive network of areas involving primary and associative visual areas, but also vestibular associative cortices that mediate the perception of body motion (vection). Our data demonstrate that this complex network of higher associative areas is established and well developed by 7 wk of age, including the vestibular associative cortex. Interestingly, the maturation of the primary visual cortex lags behind the higher associative cortex; this suggests the existence of independent cortical inputs to the primary and the associative cortex at this stage of development, explaining why infants do not yet perceive motion with the same sensitivity as adults.

## Introduction

The infant visual brain is immature at birth. To date there is no direct functional evidence from awake infants showing how the various cortical areas of human visual cortex develop. The available evidence suggests that infants are capable of discriminating motion-direction soon after birth [1–3], and that sensitivity to global-motion continues to mature slowly over the first 4–7 y in humans and 2–3 y in monkey [4–7]. The protracted development (after an early emergence) of global-motion sensitivity is attributed to late maturation of higher-level motion areas, such as temporo-occipital complex (V5/MT+) [2,8–12].

Previously it was hypothesized that the visual cortex develops in a hierarchical fashion with higher-order areas developing later, driven by feed-forward projections from previously developed lower-order cortical areas [9,13]. There is also evidence that some aspects of motion processing, such as the control of opto-kinetic eye movements (OKN), are limited by the development of subcortical relative to cortical function [14]. Brisk monocular OKN responses can be elicited in newborn infants, but only by motion in the temporal-to-nasal direction. Early development of subcortical mechanisms (probably of the Nucleus of the Optical Tectum) may mediate the eye-following response in this direction, while the directional sensitivity in the nasal-to-temporal direction, emerging later at about 10 wk, may be mediated by cortical mechanisms [11,15]. Even more peripheral factors, such as photoreceptor efficiency or myelination, may provide important constraints on some functional development. For example [16–18] chromatic and achromatic contrast sensitivities are limited by retinal immaturities, rather than by immaturity of cortical processing. This, together with evidence from infants who suffered from visual deprivation [19], suggests that the visual cortex may mediate vision very early after birth, provided that the incoming input is mature and transmits reliable visual information.

The subcortical input to associative visual cortex can undergo strong reorganization during development. In adult monkey and human, V5/MT+ input originates mainly from cortico-cortical connections and from independent konio-cellular LGN-Pulvinar projections that bypass V1 [20–22]. However, in the first few post-natal weeks, the major inputs to MT+ in marmoset monkey are a disynaptic connection from the Retino-Pulvinar projections (from the medial portion of the Inferior Pulvinar) [23]. This input may mediate the directional response of MT+ neurons observed very early postnatally. The present study examines whether the cortical mechanisms of motion processing are functional in very early infancy, and in particular aims to compare the selectivity of MT+ and primary visual cortex (V1) to flow motion to highlight a possible differential development.

To date there is no direct evidence about the functional development of the various cortical areas of human visual cortex or of their Blood Oxygenated Level Dependent (BOLD) response selectivity from awake infants, although a few studies have shown that it is feasible to record BOLD acoustic responses [24–26], or BOLD flash responses during deep anaesthesia or sleep [27–31]. Reliable BOLD activation to flashes in calcarine sulcus has been reported, but many studies found a reduction of the BOLD response to visual stimulation respect to no stimulation, particularly evident after the eighth week of age [28,30]. The origin of this negative, or more generally delayed BOLD response, is still under debate, and the issue is further complicated by the effect of the use of anaesthesia or sedation that have been shown to modulate BOLD response in human and animal models. In awake infants, studies using Near Infrared Spectroscopy (NIRS) [13,32–36], which measures signals of similar origin to the MRI-BOLD response, show evidence for positive responses in both young (around 8 wk of age) and older (4–6 mo of age) infants to visual stimulation. The discrepancy between MRI- and NIRS-BOLD responses is still unresolved and may reflect either the state of sedation, the sleepiness of infant or different types of visual stimulation. Interestingly, at 6 mo NIRS BOLD amplitude is reduced in response to homogenous flashes and increases in response to a structured high contrast pattern [34], suggesting that the type of visual stimulation matters.

Here, by measuring BOLD responses to coherent versus random flow-motion stimuli in cooperative infants, we demonstrate that the major circuits mediating motion perception are operative very early, by 7 wk of age. We found a selective response to coherent flow-motion in the temporo-occipital area, cuneus, posterior parietal and posterior associative insular cortex, with similar activation and localization as adults for visual motion [37–39] and vection perception [39–44]. Previous works have shown that resting-state connectivity networks are well segregated in newborn and even in pre-term infants [45–48] and mature rapidly in the first 2 y of age [48–50]. Here we demonstrate adult-like functional connectivity between many motion selective associative areas, but not between primary visual cortex and temporo-occipital (putative MT+) and posterior-insular cortices. The results localize for the first time cortical areas with high selectivity to visual stimuli in the first weeks of life, revealing an unexpected early maturation of the cortical system for motion processing.

## Results

We presented random-dot coherent flow-motion patterns, which cycled through radial, spiral, and contraction trajectories [37] at optimal conditions for infant vision: low temporal frequency, large dot-size, and high contrast (examples of the stimuli and the fixation of an infant are shown in the S1 Movie). We first located V1 using high contrast flow-motion versus blank, which elicited very strong responses in the occipital pole and in particular along the calcarine sulcus in both hemispheres of all infants, with a positive BOLD hemodynamic (see Table 1 and the single subject amplitude and average BOLD response in S1 Fig). This result contrasts with previous reports of negative BOLD activation to flashes in calcarine sulcus observed in sleeping or anaesthetized [27,28] infants, but is in agreement with those of Morita [30] who reported negative BOLD only in infants older than 60 d of age and with several NIRS studies [13,32–34] performed at similar ages.

**Table 1:** Position of the centre of mass, extension, and peak Z-value (Z*) for each infant (top) and adult (bottom) region of interest (ROI) in response to coherent versus random flow motion.

	**MT+**				** **	**V6**				** **	**PIVC**				** **	**Pcu-Cu**				** **	**V1 seed**					** **
**Infants**	**n**	**χ**	**ψ**	**ζ**	Z*	**n**	**χ**	**ψ**	**ζ**	Z*	**n**	**χ**	**ψ**	**ζ**	Z*	**n**	**χ**	**ψ**	**ζ**	Z*	**n**	**χ**	**ψ**	**ζ**	Z* rm	Z* b
BF	**537**	**-34**	**-59**	**-5**	**2.4**	**99**	**-12**	**-65**	**18**	**2.3**	**144**	**-25**	**-33**	**16**	**2.3**	**105**	**-7**	**-53**	**24**	**3.4**	361	1 ± 3	-69	-9	2.2	3.4
	301	28	-53	-5	3.3	305	15	-65	19	2.2	51	22	-22	15	2.2	61	6	-58	21	2.4						
CG	**97**	**-28**	**-46**	**3**	**2.9**	**109**	**-14**	**-56**	**21**	**2**	**164**	**-30**	**-23**	**4**	**3.4**	**-**	**-**	**-**	**-**	**-**	100	-4 ± 3	-59	-7	1.7	3
	378	35	-38	0	3.1	226	4	-59	18	2.3	62	35	-24	11	2.9	181	5	-47	12	3.0						
CM	**107**	**-38**	**-54**	**6**	**3**	**211**	**-24**	**-68**	**21**	**3.3**	**28**	**-32**	**-28**	**15**	**2.1**	**42**	**-11**	**-46**	**15**	**4.4**	1,120	-4 ± 5	-62	-14	4.0	4.9
	412	34	-55	2	2.9	511	14	-67	22	3.1	208	26	-30	16	2	-	-	-	-	-						
CT	**87**	**-40**	**-55**	**6**	**2.6**	**288**	**-5**	**-58**	**25**	**3.4**	**29**	**-28**	**-20**	**5**	**2.5**	**253**	**-11**	**-47**	**12**	**3.1**	38	3 ± 2	-64	-11	1.2	2.5
	337	34	-45	3	2.5	42	9	-56	17	2.1	18	23	-20	6	2	-	-	-	-	-						
DE	**410**	**-54**	**-45**	**-1**	**2.9**	**292**	**-7**	**-66**	**13**	**3.8**	**-**	**-**	**-**	**-**	**-**	**-**	**-**	**-**	**-**	**-**	414	-3 ± 4	-61	-16	2.7	3.7
	209	30	-42	6	4.5	410	13	-58	11	2.3	266	25	-17	6	2	-	-	-	-	-						
FEG	**673**	**-29**	**-56**	**-1**	**4.3**	**196**	**-12**	**-66**	**21**	**3.4**	**60**	**-25**	**-31**	**11**	**3.9**	**192**	**-3**	**-52**	**7**	**3.5**	501	-3 ± 4	-61	-6	4.5	4.4
	158	32	-52	-5	4.4	78	11	-67	20	3.3	46	24	-34	12	3.2	-	-	-	-	-						
FG	**59**	**-36**	**-46**	**-4**	**3.1**	**411**	**-11**	**-65**	**15**	**4.7**	**121**	**-27**	**-32**	**7**	**3**	**229**	**-8**	**-43**	**9**	**2.9**	123	1 ± 2	-58	-17	1.6	2.3
	472	33	-50	-5	5.3	246	6	-61	15	3.8	203	25	-30	10	3.5	-	-	-	-	-						
GC	**143**	**-37**	**-49**	**1**	**3.1**	**262**	**-9**	**-63**	**15**	**2.9**	**190**	**-29**	**-19**	**8**	**2.3**	**-**	**-**	**-**	**-**	**-**	183	-1 ± 3	-67	-16	2	2.4
	168	35	-50	3	3	370	15	-65	15	3.6	191	25	-16	10	3.3	-	-	-	-	-						
MG	**31**	**-40**	**-52**	**-2**	**2.9**	**144**	**-4**	**-62**	**13**	**2.6**	**36**	**-29**	**-29**	**11**	**2.3**	45	-4	-51	27	2.3	16	-3 ± 4	-62	-15	1.3	3.9
	73	38	-56	-1	3.1	-	-	-	-	-	52	28	-27	9	2.2	-	-	-	-	-						
ML	**236**	**-39**	**-50**	**-3**	**2.1**	**-**	**-**	**-**	**-**	**-**	**-**	**-**	**-**	**-**	**-**	-	-	-	-	-	239	-6 ± 3	-65	2	2.1	3.7
	312	30	-47	2	4.6	-	-	-	-	-	-	-	-	-	-	-	-	-	-	-						
OD	**230**	**-29**	**-58**	**0**	**4.2**	**96**	**-12**	**-68**	**19**	**3.7**	**443**	**-19**	**-26**	**10**	**2.8**						377	-5 ± 3	-58	-10	1.6	4.9
	121	31	-57	-3	3.9	286	13	-68	18	4.3	459	21	-28	13	4.3											
RE	**407**	**-29**	**-40**	**2**	**4**	**141**	**-13**	**-58**	**23**	**2.2**	**170**	**-28**	**-36**	**11**	**2.5**	**-**	**-**	**-**	**-**	**-**	246	-3 ± 5	-58	-9	3.1	4.5
	357	29	-49	9	3.8	263	23	-59	17	3.4	88	33	-33	12	2.6	-	-	-	-	-						
	**MT+**				** **	**V6**				** **	**PIVC**				** **	**Pcu-Cu **				** **	**V1 seed**					** **
**Adults**	**n**	**x**	**y**	**z**	Z*	**n**	**x**	**y**	**z**	Z*	**n**	**x**	**y**	**z**	Z*	**n**	**x**	**y**	**z**	Z*	**n**	**x**	**y**	**z**	Z* rm	Z* b
CE	**316**	**-42**	**-70**	**-2**	**3.4**	**881**	**-16**	**-83**	**36**	**5.2**	**140**	**-32**	**-35**	**23**	**4.0**	**-**	**-**	**-**	**-**	**-**	252	-2 ± 2	-83	-5	3.8	>6
	492	46	-72	-1	4.1	277	14	-86	36	5.3	43	35	-20	19	3.4	-	-	-	-	-						
CoGa	**192**	**-42**	**-69**	**-1**	**3.3**	**261**	**-10**	**-87**	**36**	**2.2**	**11**	**-37**	**-29**	**18**	**2.1**	**-**	**-**	**-**	**-**	**-**	604	2 ± 2	-84	4	3.7	> 6
	781	44	-62	3	5.5	220	5	-86	37	3.2	24	38	-29	22	2	209	23	-77	21	4.7						
CS	**143**	**-40**	**-67**	**0**	**3.3**	**364**	**-10**	**-88**	**35**	**4.3**	**221**	**-39**	**-30**	**15**	**3.4**	**-**	**-**	**-**	**-**	**-**	936	4 ± 7	-87	-11	3.1	> 6
	322	47	-64	-2	3.8	857	4	-95	26	3.3	120	39	-27	16	3.2	114	10	-75	7	3.2						
GPG	**788**	**-45**	**-67**	**-2**	**3.9**	**252**	**-16**	**-82**	**28**	**4.1**	**136**	**-37**	**-35**	**21**	**3.3**	**115**	**-16**	**-67**	**11**	**2.8**	1,884	0 ± 7	-90	-8	2.2	> 6
	298	47	-62	-5	4.5	293	13	-81	34	4.7	10	38	-23	24	2.7	-	-	-	-	-						
LVL	**723**	**-45**	**-68**	**7**	**4.9**	**1197**	**-16**	**-91**	**29**	**4.5**	**126**	**-44**	**-23**	**18**	**3.6**	**66**	**-18**	**-61**	**6**	**2.5**	851	-2 ± 6	-91	-5	3.9	> 6
	586	47	-67	1	> 6	1234	14	-89	29	> 6	143	43	-24	14	4.9	291	8	-62	4	3.2						
MCM	**387**	**-39**	**-69**	**-2**	**3.5**	**376**	**-7**	**-82**	**29**	**4.4**	**301**	**-43**	**-34**	**18**	**4.3**	**433**	**-5**	**-66**	**13**	**4.9**	3,998	-2 ± 6	-88	-12	3.3	> 6
	1147	41	-64	-5	> 6	740	7	-88	26	> 6	313	39	-31	19	5.5	115	8	-70	3	3.3						
MM	**128**	**-42**	**-56**	**4**	**3.7**	**122**	**-7**	**-90**	**28**	**2.7**	**57**	**-34**	**-26**	**18**	**3**	**-**	**-**	**-**	**-**	**-**	1,107	2 ± 2	-86	-6	>6	> 6
	189	41	-52	-5	4.2	267	7	-89	28	4.2	34	34	-22	15	2	307	1	-53	13	2.2						
PA	**287**	**-49**	**-62**	**1**	**2.7**	**943**	**-7**	**-78**	**32**	**3.2**	**139**	**-40**	**-29**	**15**	**3.3**	652	-1	-62	18	4.6	2,022	4 ± 7	-84	-3	3.5	> 6
	580	-39	-60	0	2.9	1007	8	-79	30	4.9	325	41	-27	16	3.1	-	-	-	-	-						
PE	**545**	**-48**	**-60**	**-3**	**3.9**	**1118**	**-15**	**-89**	**27**	**5.4**	**222**	**-38**	**-29**	**15**	**2.1**	893	-4	-70	30	4.3	763	-1 ± 5	-85	0	3.6	> 6
	994	51	-59	3	5.3	1287	17	-86	27	5.3	126	42	-29	13	3	-	-	-	-	-						

Analysis was performed using a General Linear Model (GLM) approach for each single subject with a statistical threshold of α = 0.05 (corresponding to a minimum Z-score value of 1.96). The coordinates are in millimetres from the anterior commissure (AC) point for infants (“χ, ψ, ζ”notation) and in Talairach space for adults (“x, y, z” notation). Note that V1-seed ROI is bilateral and the V1 Z-score related to the response to coherent flow-motion versus blank is reported in the column Z*b; the Z-score of the response to coherent versus random flow-motion is reported in the Z*rm column and many of these values are not statistical significant (<2). Bold rows refer to regions in the left hemisphere. (MT+: temporo-occipital complex; V6: Visual area six; PIVC: posterior insular vestibular cortex; PCu-Cu: PreCuneus-Cuneus; V1: primary visual cortex.)

**Fig 1:**
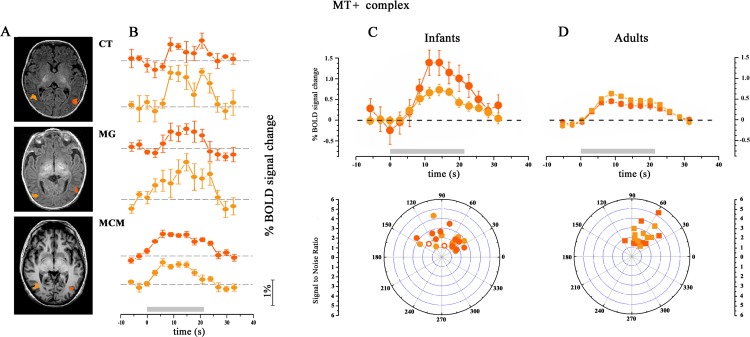
Localization and BOLD responses of complex MT+. A: Example of localization of complex MT+ in two infants (CT and MG) and one adult (MCM; bottom row) in response to coherent- versus random motion (threshold at *p* ≤ 0.01). Images in radiological convention. B: time-courses of the individual ROI in A for the right (light orange) and left (orange) hemispheres, averaged over 4 and 5 periods for CT and MG respectively. The grey bar indicates the duration of coherent motion. C- top: Time-course averaged across all infants for the right (light orange) and left (orange) hemispheres of MT+ (bars ±1 standard error of the mean [sem]). C-bottom: reliability of responses of individual infant MT+. The distance from the origin indicates the Signal to Noise (S/N) ratio evaluated as the ratio between the amplitude of the fundamental frequency to the mean square root of the amplitude of the adjacent frequencies. The angle reports the phase of the fundamental frequency response (in degree). A phase of 64° corresponds to a standard hemodynamic delay. D. Same convention as C for adult subjects. Filled symbols correspond to *p* < 0.01, open to 0.01 < *p* < 0.05 threshold. Note that all infants’ responses have a S/N ratio greater than 1. (MT+: temporo-occipital complex; s: seconds.) Numerical data are available in the S1 Data, Fig 1.

Using this calcarine activation (labelled V1 seed), we generated a mask comprising all voxels that correlated positively or negatively with the labelled V1 activity (S2 Fig). To allow for variability in the hemodynamic time-constant demonstrated in previous studies [24,25,27,28,51], we computed the correlation using the delays between 0 and 3 s, after verifying that using additional delays would not change significantly the final labelled regions of interest (ROIs) (see Methods for details). Within this functional mask, we studied significant response modulation (positive and negative) to alternation of coherent- against random-motion stimuli, constructed to match locally the motion velocities of the coherent flow stimulus. The motion difference between the two stimuli is very subtle, but in adults it is able to elicit a consistent BOLD activity in a network of areas [37–39], similar to that we observed in infants. Fig 1A and 1B shows examples of the responses of two infants (CT and MG, *p* < 0.01 uncorrected) for a temporo-occipital cortical area that strongly preferred coherent to random flow motion, both in the right (light orange) and left (dark orange) hemisphere.

Both the anatomical localization (close to the inferior-temporal sulcus as assessed by an expert neonatal neuro-radiologist), and the time-course of the infant responses, were very similar to those of adults using similar statistical thresholds and procedure (see Fig 1A and 1B, subject MCM, *p* < 0.01). Table 1 reports the location and extension of these areas for all subjects. The adult V5/MT+ was clearly labelled in a location similar to previous studies [37]. Despite the great difference in atlas and morphometry between adult and infant brains, the coordinates of these areas were highly consistent between the two groups of subjects [37]. Given the similarity in anatomical localization (for congruency between localization in infants and in adults see multi-subject section) and the response selectivity to coherent flow-motion between adult and infants, we labelled this area as the putative infant MT complex (MT+). In all infants, the responses of MT+ were reliable at a threshold of *p* < 0.01 in 22 out of 24 ROIs. For only two MT+ ROIs, the statistical threshold had to be reduced to *p* < 0.05 before activity of similar cluster-size could be identified at the expected anatomical location. An independent measure of reliability is given by the Signal to Noise (S/N) ratio, which measures the relative power of a modulated signal at the fundamental frequency of the stimulus repetition respect to close-by frequencies. Scalar S/N values were similar to those measured in adults (Fig 1, compare C with D). Also the phases of the responses, that in principle could vary uniformly in the 0–360° range, were tightly clustered in infants and adults and the vectorial statistical analysis between the S/N response of infants and adults (Left and Right MT+ pooled together) showed that the BOLD responses were not significantly different (See S1 Table). Also the differences between Left and Right hemisphere BOLD responses averaged across subjects (see Fig 1C and 1D top panels) were not significant at either age. However, the variability across infants is somewhat larger than between adults, but this is to be expected given the higher level of measurement errors, including the size of stimulated visual field, eye movements and age range.

**Fig 2:**
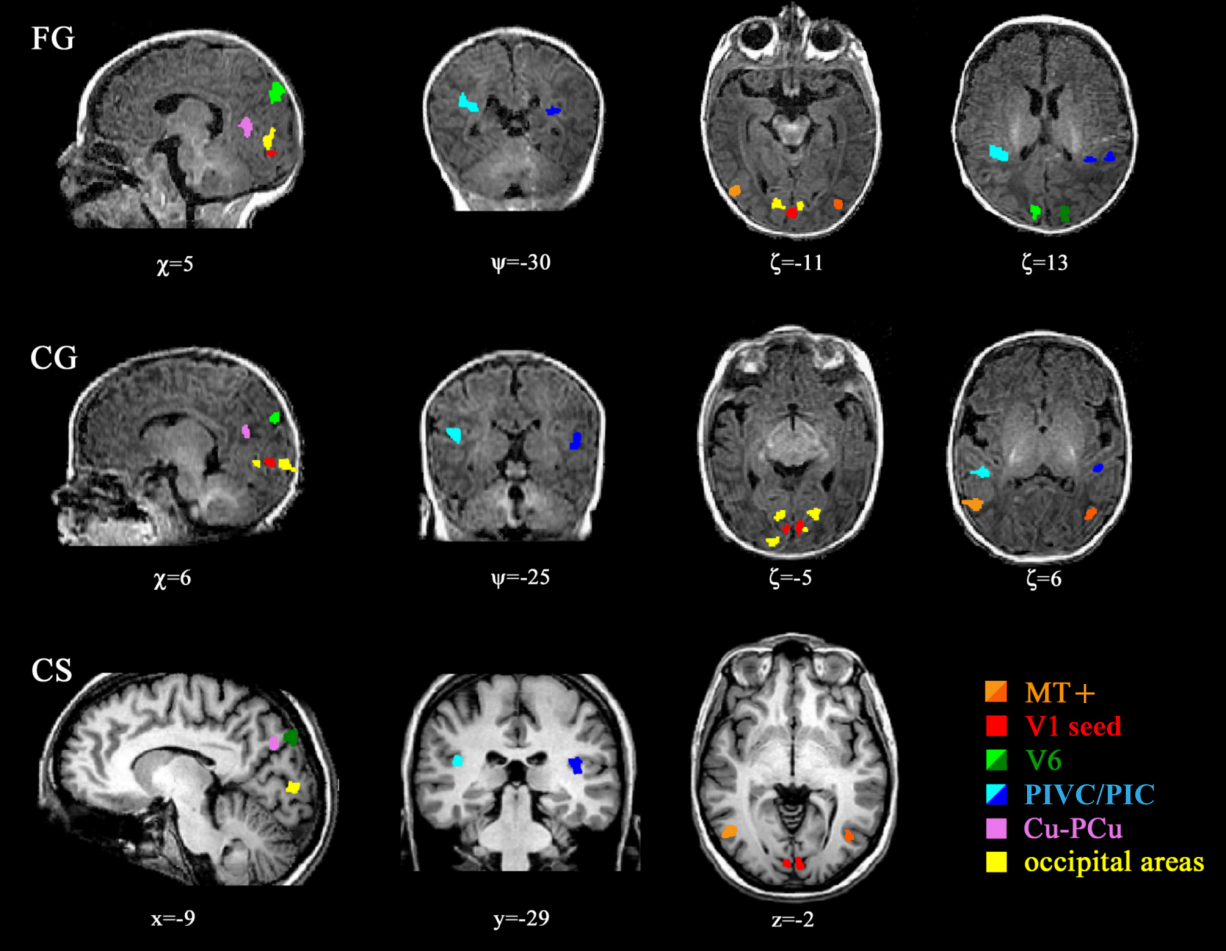
Localization of the motion-network areas. Example of localization and extension of all major areas (ROIs) in two infants (FG and CG) and one adult (CS, bottom row). Light colours for right hemispheres, dark colours for left hemispheres. The location of each bi-dimensional section is indicated in millimetres from AC point for infants (“χ, ψ, ζ” notation) and in Talairach coordinates for adults (“x, y, z” notation). Note that the V1 seed was bilateral and has been labelled on the response to motion versus blank (V1 seed). Several occipital areas labelled in yellow are not included in the detailed analysis, given the difficulties in distinguishing between them in infants. (V1: primary visual cortex; MT+: temporo-occipital complex; V6: Visual area six on medial-parieto-occipital region; PIVC/PIC: posterior insular vestibular cortex and posterior insular cortex respectively; PCu/Cu: Pre-Cuneus/Cuneus.) Numerical data of size and localization are reported in Table 1.

**Fig 3:**
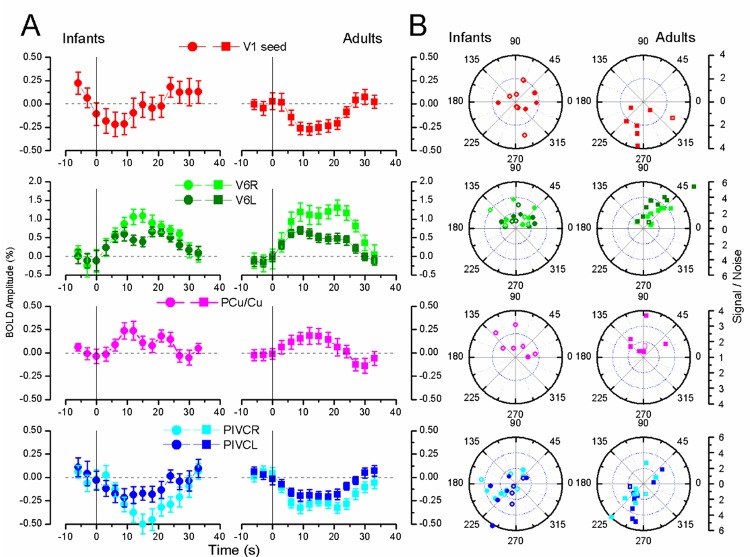
Average time-course and individual Signal to Noise ratio of the motion BOLD response. A: Average time-course for V1 seed, V6, PCu/Cu, PIVC/PIC in infants (left) and adults (right) in response to coherent versus random flow-motion (threshold at p ≤ 0.05). Light colours refer to the right hemispheres ROIs, darker colours to the left. Bars represent ±1 sem. Infant and adult responses are very similar. B: Signal to Noise ratio polar plot for individual subjects (left column infants, right column adults). Convention as in Fig 1C and 1D. The points form clear clusters indicating that the response is reliable. For only the V1 seed did two infants have S/N less than 1. Note that the scale in A for V6 ROI is compressed, given the larger activity of this area. Filled symbols correspond to *p* < 0.01, open to 0.01 < *p* < 0.05 threshold. (V1: primary visual cortex; MT+: temporo-occipital complex; V6: Visual area six on medial-parieto-occipital region; PIVC: posterior insular vestibular cortex; PCu/Cu: Pre-Cuneus/Cuneus.) Numerical data are available in S1 Data, Fig 3.

The network of regions analysing flow in adults is very extensive, comprising (besides V5/MT+) many dorsal associative visual areas (V3, V3A&B, TOS, LO) [37,52], as well as multimodal cortices such as V6 [40,41], Pre-Cuneus, cingulate sulcus and an associative vestibular cortex located in the posterior insular cortex, PIVC/PIC [39,41,43,44]. Responses to coherent versus random stimuli were observed in all adults, both in the dorsal visual and the associative multisensory cortices of this network (Fig 2 reports the anatomical location of the labelled regions in adult CS and Table 1 the Talairach coordinates of their foci, with the peak Z-score and the size of labelled ROIs at *p* < 0.05). Nearly all these areas, particularly V6 and the Cuneus/Pre-Cuneus areas, showed a strong positive preference to flow-motion, while the vestibular cortex PIVC/PIC and V1-seed (the ROI selected in response to coherent motion versus blank) showed a positive preference for random motion, consistent with previous reports in adults [40,52]. Not only are the anatomical locations of these areas very similar in adults and infants (see Table 1), but so also are the BOLD modulation (Fig 3A), the reliability and response delay (S/N and phase respectively, Fig 3B). Only minor differences emerge. The V1-seed ROI, selected by the response to flow-motion against blank, showed a significant negative response in adults to coherent versus random flow-motion (two tailed *t*-test, t = 2.70, *p* < 0.02), but not significantly different from zero in infants (two tailed *t*-test, t = 0.65 *p* = 0.47), indicating possible delayed development. While it was not possible to compare BOLD amplitude of the ROIs between infants and adults, given that the ROIs were defined using the same dataset (statistical circularity), it was possible to compare delays. The responses of V6 (ɸ(infant) = 82 ± 40 °; ɸ(adult) = 55 ± 31 °) and PIVC/PIC (ɸ(infant) = -146 ± 35 °; ɸ(adult) = -117 ± 38 °) regions had a slightly larger hemodynamic delay indicating a delayed BOLD hemodynamics in infants, in agreement with previous results in different cortical area [25,51] (S1 Table reports the significance tests calculating by vectorial statistics). Interestingly the infant delays with respect to adult delays vary between areas and between stimuli. For example, the response of the MT+ and V6 ROIs to flow versus blank is negative in some infants (see S3 Fig), suggesting a specificity of the Negative BOLD effect for the visual stimulus [34].

Besides the ROIs reported in Table 1, many other occipital areas were labelled in the individual infants. We did not analyse in detail these associative occipital cortices (yellow in Fig 2), given that these regions often blurred with each other (because of their close proximity in the small infant brain) and are very difficult to classify by anatomical landmarks.

### Multi-subject Analysis

**Fig 4:**
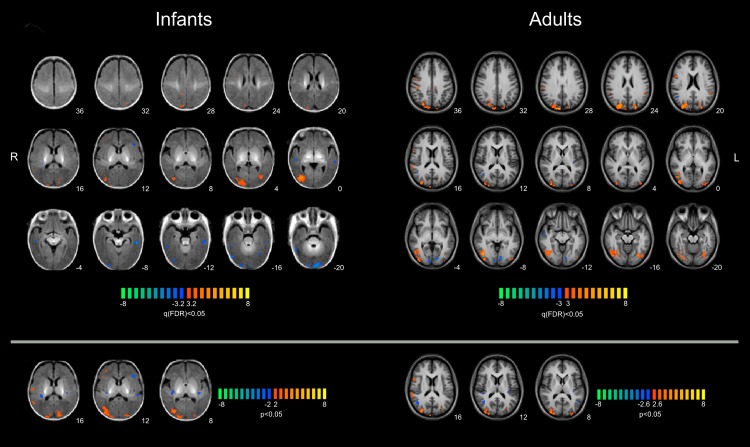
Multi-subject GLM analysis for coherent versus random flow motion stimuli. Results of a multi-subject fixed-effect general linear model (FFX-GLM) analysis for the BOLD response to coherent versus random flow motion performed on the group of 11 infants and registered on the infant brain template on left panels and on 9 adults in the right panels. Colour bar represents the statistical t-values, with thresholds corresponding to a q(FDR) < 0.05 in the top panels and to *p* < 0.05 uncorrected in the bottom panels. Numbers refer to the “ζ” coordinate of the transversal slice for the infants and to the Talairach z for the adults. R-L in radiological convention. (R: right; L: left; FDR: false discovery rate.) Data of the statistical maps are available in S2 Data.

The flow-selective area in the Cuneus/Pre-Cuneus was labelled in only seven infants, while it was always present in all except one adult; in another subject, the V6 and PIVC/PIC ROIs were not significant at α = 0.05. To assess the congruency of ROI localization, we built an anatomical template by brain segmentation using dedicated tissue probability maps (illustrated in S4 Fig) and we performed a fixed-effect GLM multi-subject analysis (see Methods). Fig 4 (top panels) shows the results mapped on the same anatomical template of S4 Fig for infants and in Talairach atlas for adults, at the same statistical threshold of q < 0.05 (false discovery rate [FDR] corrected and without mask). In adults, the occipital foci associated both with dorsal and with ventral pathways [53,54] showed a stronger response to coherent motion, with the exception of V1/V2 areas. A negative response was also labelled in the posterior insular cortex of the left hemisphere (slice at z = 8), while the right PIVC/PIC became labelled lowering the threshold at *p* < 0.05 (lower panels). This result is consistent both with the large variation in phase of the PIVC/PIC responses (see Fig 3 and S3 Fig) and with the large variation in the localization between subjects (see Table 1). The variation in phase was to be expected given that the BOLD amplitude of PIVC/PIC is modulated by the strength in vection perception [39–43]: subjects with stronger vection illusion might show stronger negative responses. Similarly, also the large scatter in position of the PIVC/PIC region has been already reported [43]. Both factors indicate that the multi-subject GLM may not be the most suitable technique to locate this area. Nevertheless it is reassuring that when decreasing the threshold to *p* < 0.05 (bottom panels) only one clear additional cluster in the posterior insular cortex became labelled, reinforcing the suggestion that PIVC/PIC localization can vary considerably across subjects. In infant multi-subject GLM, no activity was significantly labelled for V1/V2; all the ventral areas along the fusiform and the lingual gyri had negative responses, while the adult responses were positive, indicating a late development of direction selectivity for the ventral pathways. In contrast, the dorsal areas, like V3/V3A, LO, TOS, V6/V6A, and MT+, were clearly labelled, and showed a preference for the coherent flow motion. In the right hemisphere (slice at ζ = 16) a negative response corresponding to the location of the PIVC/PIC was labelled. As in adults, decreasing the threshold at *p* < 0.05 (bottom panels) also the PIVC/PIC in the left hemisphere became detectable, suggesting that, as in adults, for infants, the localization variability of this area is high. There were also other negative and positive clusters in the temporal lobe (see for example slice at ζ = 0) that can be also observed in adults, but whose function and circuitry are still unknown. The similarity in the localization pattern between infants and adults strongly suggests that the development of visual primary and dorsal associative cortex for motion processing is quite well advanced by 7 wk of age.

**Fig 5:**
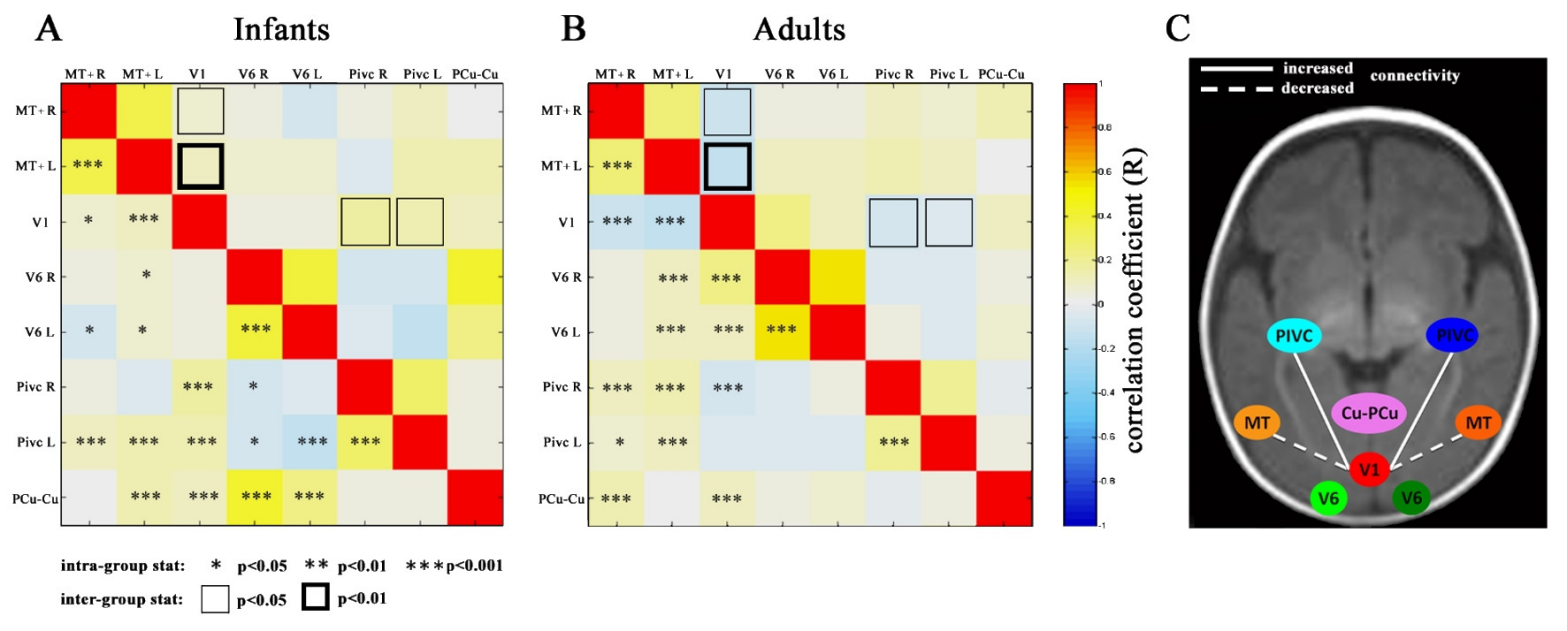
BOLD resting state network. A and B: Correlation matrices of the BOLD resting state activities between all possible ROIs in infants (left) and adults (right). Each value (see scale) shows the average of the correlation across subjects. The significance of each value is indicated by the stars in the checks below the main diagonal, showing the p-values obtained by the cross-correlation calculated concatenating the normalized resting state BOLD signals of all subjects of the group. To test for differences between infant and adult correlation matrices, a two-tailed *t*-test was performed between the two groups, and the checks with contour above the main diagonal indicate the two significantly (at *p* < 0.01 and *p* < 0.05) different values. C: The diagram illustrates the two correlations that are different between adult and infants. The weak connectivity in infants between V1 and temporal-occipital areas was also observed in [46,48]. (V1: primary visual cortex seed; MT+: temporo-occipital complex; V6: Visual area six on medial-parieto-occipital region; PIVC: posterior insular vestibular cortex; PCu/Cu: Pre-Cuneus/Cuneus; R: right; L: left.) Numerical data are available in the supporting file “S1 Data“, Fig 5.

In nine out of twelve infants, we were also able to record resting-state activity during spontaneous sleep. Fig 5 shows the correlation of the resting-state activity between each pair of the ROIs of Fig 2, for all possible combinations in infants and adults. In most regions of infants and adults the correlation was strong and significantly non-zero (indicated by stars in the individual cells in Fig 5). Significant correlations reflecting functional connectivity were present in both adults and infants between homologue areas of the two hemispheres, including PIVC/PIC, V6 and MT+. The correlation matrixes were similar in adults and infants, corroborating the similarity of the BOLD responses with two main exceptions. In infants, the correlation between bilateral V1 activity and MT+ was significant (although just marginally for the right hemisphere), but it was positive, while in adults the correlation was strong and negative, as previously reported [55,56]. Similarly, the PIVC/PIC activity in infants showed a significant positive correlation, while in adults it was negative.

## Discussion

Our results show that visual motion cortical areas have a high degree of functional specialization in very young infants: direction selectivity is well established in many dorsal cortical regions at 7 wk of age. To distinguish between coherent and random flow-motion, the neuronal mechanisms must be selective to motion direction and also integrate local-motion signals along complex trajectories. Direction selectivity in adult primates occurs initially in V1, while large spatial integration along complex trajectories occurs in V6 and V5/MT+, and propagates to higher visual cortices [42]. Given that 1-mo-olds (or younger) infants show defensive motor responses such as blinking and avoidance head movements in response to large-field radial expansion patterns [1,3], and that motion direction selectivity has been demonstrated behaviourally [2], it is reasonable to assume that selectivity for motion direction emerges around 7 wk of age or earlier in many regions of the dorsal pathways, including MT+ and V6, both crucially important for motion perception. This suggests that cortical processing of motion is more mature than has been proposed at this age [8,11,15], with a relatively stronger response of MT+ compared with occipital areas in infants. Interestingly, V5/MT+ is situated ventro-laterally, just posterior to the meeting point of the ascending limb of the inferior temporal sulcus and the lateral occipital sulcus, and corresponds, on individual subjects, almost precisely with Flechsig's Field 16 [57], one of the areas that is myelinated at birth, corroborating the suggestion that V5/MT+ should be considered as a primary sensory area with an early maturation [58]. An earlier maturation of the V5/MT+ is also consistent with ERP evidence at 5 mo of age of a more lateralized selectivity to coherent motion in infants with respect to adults [59], indicating that the delayed maturation of more occipital areas (with respect to V5/MT+) is protracted over several months. Considering that in adults there is evidence that direction selectivity is computed in MT+ rather than being inherited from inputs of earlier cortical regions [22,60], it is feasible that the MT+ directional selectivity BOLD response at 7 wk of age is also computed locally, and it is independent from V1 input. The neuronal processing of the ventral and dorsal visual pathways are quite distinct and mediate different visual functions [53,54]. Ventral area, like V4 and fusiform gyrus, showed preference for coherent motion in adult, although these areas are not considered crucial for the perception of motion direction and probably inherit the direction selectivity from dorsal areas. Interestingly in infants, these areas prefer incoherent motion as illustrated in Fig 4. These results suggest that the early development of cortical specialization might be a prerogative of the dorsal pathway and visual motion analysis, corroborating the evidence of a later maturation of the ventral stream in children [61] sub-serving object and face recognition [62,63].

Overall, our data suggest that both primary and dorsal associative cortices develop direction selectivity at a similar stage. The motion selectivity of V6-, Pre-Cuneus and associative vestibular cortex has been related to the illusory percept of body-motion induced by large flow field (vection) [39–41,43]. The similar activity of these regions in infants and adults suggests that infants may have a sense of vection and hence a sense of body position. The continuous stimulation of the semi-circular canals in the womb may induce early development of the vestibular system, so the few weeks of visual experience may be sufficient to endorse the vestibular-visual integration necessary for vection perception. While this is consistent with early development of the vestibulo-ocular response in newborn infants [64], it is surprising that visuo-vestibular cortex is selective to motion so early, as optimal multisensory integration develops slowly in humans [65–67].

Our data show that the hemodynamic response of infants is delayed with respect to adults, similar to that reported at similar ages in response to acoustic [25] and motor/tactile stimulation [51]. The delay of the response is of the order of few seconds, consistent with the delay observed by Arichi [51], and cannot explain the phenomenon of Negative BOLD corresponding to 180 degree of phase shift and delay on the order of tens of seconds. It is worthwhile noting that other laboratories have observed positive BOLD visual responses in this age window, and negative BOLD responses for older infant [30], reinforcing the suggestion that the Negative BOLD phenomenon is related to the functional maturation of the cortex. However, the fact that NIRS studies in awake infants consistently find a positive BOLD responses at all ages [13,32–36] suggests that the MRI-BOLD negative response may not result from synaptogenesis or the cerebral metabolic rate for oxygen, but rather from the alertness state of the infant. In addition, the positive versus negative BOLD response could be biased by the use of flash stimuli versus black background, rather than a spatial pattern whose contrast is modulated over time. This is supported by the finding that the phase of V6 and MT+ became negative in response to the flow versus blank (S3 Fig), and blank stimuli engage usually little attention in infants. Interestingly, a recent NIRS study showed a negative BOLD response to flashes and a positive BOLD response to high contrast flickering patters in 6-mo-old infants [34]. It is well known that flash stimuli are strongly subject to suppression during blinks, and the suppression may be even stronger when the eyes are shut or during sleep. Repeating our study with older collaborative and awake infants could provide important clues to resolve the origin of negative MRI-BOLD response in infants, although the difficulty in recording stable signal increases rapidly with age because of the increased motility of older infants.

During post-natal development, there is a continuous refinement of anatomical connections in the mammalian visual system, which are initially diffuse and then progressively pruned to increase target selectivity. Between 0 and 4 mo, synaptic density increases rapidly [68,69], and myelination intensifies along the visual pathway. Diffusion Tensor Imaging (DTI) studies suggest that at birth all major fiber systems are in place, despite low anisotropy value [70] and incomplete myelination [71]. The functional connectivity observed in our data showed an adult-like correlation between inter-hemispheric ROIs and between associative cortices, in agreement with the anatomical DTI findings of well-established fiber connections. However, connectivity between V1 and MT+ and PIVC/PIC were different than in adults. In infants, the optic radiations are diffuse (see for example Fig 3 in [72]) and may project not only to V1 but also to other visual cortices. In addition, other transient projections not originating from LGN may be functional in young infants. These diffuse afferent projections to the cortex might mediate the strong BOLD response observed here in all areas, without implicating adult-like cortico-cortical connections, which at this age are highly immature [69]. This model would also explain the weak (although significant) correlation between MT+ and V1, given that these areas would receive independent V1 inputs to generate the strong BOLD response selective to motion. It would also reinforce the suggestion that, in infants, BOLD directional selectivity is computed in MT+ rather than being inherited from inputs of earlier cortical regions. However, some caution is required in comparing adult and infant functional connectivity given that the infant, but not the adult, data were acquired during sleep. Several studies show that functional connectivity during sleep and relaxation [73] may be different, but the differences are not significant for the sensory-visual resting state networks [74]. Resting-state functional connectivity appears to be invariant even under anaesthesia [75].Therefore, we do not foresee that different depths of state of alertness would affect the outcome of our studies. In addition, most of the functional connectivity strengths are equal in infants and adults, in agreement with previous results [48]: only V1-MT+ was very different. It would be rather strange for sleep to affect so selectively only this connectivity.

As in human, in cat and monkey, the putative corresponding area of human MT+ is well developed at birth, with a developmental time-course similar to primary visual cortex [57,58]. V5/MT+ neurons of newborn monkey show directional selectivity [8]. Interestingly, in monkey it has been suggested that the fast maturation of V5/MT+ is mediated by strong and direct retino-pulvinar-cortical projections, which are later pruned during development [23]. In adult humans the LGN-MT+ projections, which bypass V1, probably overtake the functional role of this direct retinal-pulvinar input [20–22], helping to explain several motion abilities retained after lesion of V1 (such as “blindsight”). The existence of a direct retino-pulvinar input to MT+ also in human infants would explain the weak functional connectivity between MT+ and V1. However, other explanations cannot be excluded, including delayed development of feedback projections, which may have an overall suppressive effect, explaining the negative correlation in adults. The suppression of V1 activity by feedback connections would also be consistent with the negative BOLD response to motion (against noise) of V1 in adults, and the smaller negative (not significant) modulation in infants, suggesting an overall delayed development of V1 circuitry respect to MT+.

Whatever the anatomical connections between the various areas in infants, the extensive, well-developed network of visual associative areas selective to motion at 7 wk, demonstrated by this study, suggests that direction selectivity, the most fundamental property for motion perception, emerges very rapidly, and simultaneously in many dorsal cortices. This developmental pattern may be consistent with the early suggestion [76] that direction selectivity development is limited by peripheral factors such as myelination and speed of neuronal signal transmission. Timing of arrival of spike input to cortex is crucial for motion perception. Jitter in temporal delay of the order of milliseconds is sufficient to disrupt or even invert motion [77]; disorganization of input spike trains or high levels of noise may be sufficient to impede the formation of direction selectivity at the cortical level. As input motion signals become more reliable and organized, the cortex may be capable of developing the complex neuronal circuitry for direction selectivity and trajectory integration only within a very limited time window. Similar fast cortical development has been observed previously for stereo acuity or chromatic contrast (for review see [76]), and also for these functions, the limiting factors may be peripheral. If the present suggestion of independent emergence of direction selectivity in MT+ is confirmed by additional evidence, we should revise the widely accepted idea of a slow, uniform, and progressive maturation of the various cortical hierarchy levels [9] and of cortical motion responses [59]. We propose an alternative model in which all dorsal cortical regions have equal potential for fast maturation and for developing appropriate circuitry, once the input begins to transmit reliable neural information.

## Methods

The study was approved by Ethics Review Board of Fondazione Stella Maris and Regional Paediatric Committee (Meyer Paediatric Hospital). Written informed consent was obtained by all subjects or their parents before the experiment.

Sixteen (five females and eleven males) healthy, full-term, awake infants, mean age 7.7 ± 1.2 wk (range = 6.6–10.4w), were scanned by a 1.5T MR scanner (GE Healthcare, USA). Data from three subjects were not included in the analysis because of movement artifacts or deep sleep, and for one subject we obtained only the anatomical dataset, leaving twelve subjects with full data. All infants were assessed by an expert paediatric neurologist by means of the Hammersmith Neurological exam [78] and a battery of visual function tests, including fixation to white and black targets and the ocular following response. The infants were again assessed at 3 mo with a neurological follow-up that confirmed normal development. After the MR exam and before any data analysis, an expert child neuro-radiologist performed also an examination of the anatomical scans to reveal possible anomalies and all infant brains were referred as normal.

The MR protocol comprised acquisition of a three-dimensional (3-D) T1w FSPGR sequence (TR/TE = 12.28/5.14, isotropic voxel = 1x1x1mm^3^), and an fMRI session of three different series (GRE-EPI, TR/TE = 3000/50, FA = 90°, FOV = 240 X 240 mm, matrix = 96 X 96, slice thickness = 3 mm). fMRI experiments included two series of 84 time points (4’12” duration, block design, six periods of alternating coherent flow motion versus a blank or a random motion condition, each lasting 21 s). In nine subjects (age range = 6.6–8 wk) a resting state fMRI series (120 time points, 6’00” duration, no stimulus presentation) was successfully acquired during the spontaneous sleep. All functional series were preceded by four dummy time points to allow signal stabilization. Stimuli were generated in Matlab (TheMathWorks) and displayed on LCD goggles (Resonance Technology) positioned inside the head coil 10 to 15 cm from the infant eye, giving a visual field of about 27 X 20 degrees.

Infant eyes were refracted with retinoscopy at a distance of 87 cm (the virtual image of the goggles is greater than 1 m). All infants were in the normal range between 0 and +2D: given the viewing distance, no additional correction was introduced. Fixation of infant gaze was monitored by an infrared camera installed within the goggles (sample frequency 60 Hz, see final segments of S1 Movie for fixation example). Usually infants scanned the stimulus very attentively and this, together with the real-time movement signals, gave an indication of their level of wakefulness. Infants entered the magnet lying down on the scanner table, swaddled in a sheet by an expert neonatal nurse to calm the baby and to reduce movement. One operator (MCM, SAC, or LB) accompanied the child into the magnet, taking the sphinx position, surrounding the body of the child with her arms and wrapping his/her head with her hands in order to maintain a physical contact, to facilitate calming and to reduce motion. The operator was in constant communication with the staff at the acquisition terminal. Infant ears were protected by cotton wool padding at the auditus of the external auditory canal, and sound-attenuating headphones. In order to reduce the stress of the babies, we adopted specific strategies in accordance with the character and routines of each individual child. Some preferred to use a pacifier to relax and to sleep, especially for anatomical and resting state fMRI scanning. If the infant moved or changed position the operator could reposition the goggles by tilting them. In these cases, the recording was continued, but the series was cut offline (see details below). Importantly, the operator could not see the visual stimulus delivered, or if the goggles were switched off for the resting state scan. During the anatomical scan, the goggles were switched off and nine of the infants fell asleep, allowing us to acquire the resting-state scan.

The same MR protocol described for children was repeated in nine healthy adults (six females and three males, mean age 35 y). In adult subjects, resting state scans were acquired by asking to the subjects to relax and stay still with eyes closed.

The visual motion stimuli comprised 100 dots, half black and half white, of 1.3 degrees diameter, moving at constant speed (5 deg/s) with limited lifetime of 10 frames (at 60 Hz about 160 ms). For coherent motion the trajectory of the dots changed gradually from expansion, inward-spiral, rotation, outward-spiral, contraction, then repeating the cycle again (see S1 Movie, initial segments). The full cycle lasted 2 s. The random motion was constructed using the same dot velocities shuffled randomly over the dots, so it had matched local motion power. The dots covered all the visual field except the central 2 degrees. Dot density was kept constant in all displays, and collision between dots was not allowed. The mean luminance was 20 cd/m^2^ and contrast 0.85 (for further information, see [37]).

Three-dimensional T1-weighted images of each infants were visually inspected to select good quality data in order to create a specific infant template for this study. Only one infant’s data (subject DE) was discarded because of the poor grade of anatomical images; all the remaining twelve 3-D datasets were selected and analysed with the SPM8 package with the Diffeomorphic Anatomical Registration Through Exponentiated Lie algebra (DARTEL) algorithm [79]. DARTEL uses diffeomorphic warping to obtain a study-specific template, with an optimal inter-subject realignment and an improved co-registration of small structures, such as those of infant brains. A pre-conditional step for the use of this algorithm is the segmentation of brain tissues using standard tissues probability maps. For infants, we used the segmented partial estimate volumes for grey matter, white matter, and cerebral spinal fluid derived by the 36 MRI dataset of 3-mo-old infants available at http://jerlab.psych.sc.edu/NeurodevelopmentalMRIDatabase/index.html as tissue probability maps [80]. The obtained atlas is shown in S4 Fig.

Data analyses were performed with BrainVoyager (BV, Brain Innovation). First, for each functional series, motion spikes or periods of heavy movement that could not be compensated for were eliminated, and the resulting separate intervals were considered as independent shorter samples. Infant motion was estimated by calculating the six rigid body parameters (three for translation and three for rotation) across time. Time series with head motion greater than 4 mm (translation) or 5° (rotation) were excluded. For each subject and stimulus, the mean and maxima values of the six rigid body parameters registered in the survived (and used) time courses are reported in S2 Table.

The recorded eye movements were analysed to verify fixation and alertness of the infant; intervals of sleep or jerk movements that could not be adequately compensated were discarded from analysis, typically for the duration of half period of stimulation. For all infants, we were able to select at least half duration of the complete recorded run (on average, 4.9 ± 0.8 periods for coherent versus random flow motion, 4.8 ± 0.9 periods for coherent flow motion versus blank, and 110 ± 10 data points for resting state stimulus) for further analysis, with the remaining periods discarded due to head motion or sleep. Data preprocessing included mean intensity adjustment to compensate for interscan intensity differences, temporal interpolation and re-sampled to compensate for slice dependent time differences (sinc function), 3-D motion correction (sinc interpolation), and high-pass temporal filtering (GLM-Fourier approach, two cycles).

Functional data were co-registered on the three-dimensional anatomical T1-weighted images by using an affine alignment with the standard BV nine parameters (three for translation, three for rotation, three for FOV scale). Infant anatomical datasets were in turn transformed into the AC-PC coordinate system by applying a rigid transformation (6 parameters; 3 for translation and 3 for rotation), whilst adult data were transformed into the standard Talairach space. For each subject, BOLD responses were analysed using a GLM modelling the regressor of interest (by convolving a box-car function for each stimulation block with a gamma variate function for the hemodynamic response) and six spurious movement-regressors (outputs of the 3-D motion correction procedure).

The first stimulus (flow-motion versus blank) was used to select a bilateral region with very strong response, located along the left and right calcarine sulci (*p* < 0.001 in infant and *p* < 10^−10^ in adults). Many other strong activities in the occipital pole were present but not analysed. The signal registered in this ROI (labelled V1-seed) was used to calculate correlation maps by cross-correlating the V1-seed ROI signal with all other brain voxels, using temporal delays in the range 0 to 21 s, given the unknown hemodynamic delay of infant BOLD responses. S2 Fig shows the maps of four different infants computed for positive and negative correlation at zero delay and at *p* < 0.05. The low conservative threshold of *p* < 0.05 marked about 40% of all voxels for delay = 0 s and additional 26% for delay = 3 s. Computing the maps for the remaining delays in the range of 3 to 21 s added only about 30% new voxels. Repeating the same analysis only on the selected ROIs (see below the selection procedure), we obtained that 80% of the voxels were labelled at delay = 0 s and 94% combining delay 0 s and 3 s. Given the high proportion of labelled voxels, in the analysis presented here we applied a mask obtained by the union of those computed at 0 and 3 s delays.

Both for infants and adults, the GLM analysis of the coherent versus random motion was performed within this mask, and all positive or negative foci at a threshold of *p* ≤ 0.05 were labelled (see Table 1 for statistical thresholds of the ROIs). These ROIs were located by a neuro-radiologist (with more than 20 y of experience with newborn infants) along the major infant sulci and gyri. In adults, we restricted the analysis to the major regions of interest selective to motion [37–39] that could be easily traced in infants.

Table 1 reported the maximum foci localization in millimetres in the AC-PC coordinate system for infants and in Talairach space for adults, and the respective Z-score for areas that were identified in all adults and in most infants. For multiple foci areas, the coordinates corresponded to the positions of the centre of mass of the aggregate ROIs. In particular, for the occipital activities we reported the union of the bilateral activations of V1 (V1-seed) (reporting the average coordinates of the two foci); an area located dorsally just posterior of the parietal-occipital sulcus, which in adults correspond to the union of V6 and V6A; an area positioned posteriorly in Pre-Cuneus/Cuneus, close to the parieto-occipital sulcus and anterior of the most extreme periphery of V1/V2. This area could be the human equivalent of the pro-striate [81], not to be confused with the Pre-Cuneus selective motion area reported by Cardin et al [39,82]. Many other occipital activation sites were labelled in response to coherent versus flow motion in the mask, but given the difficulties in distinguishing between them and locating them in all the sulci, we decided not to report or analyse them in detail. The area MT+ was the easiest to locate along the inferior temporal sulcus, both in adults and infants. In adults, this ROI includes MT and MST. An area showing a differential response to coherent versus incoherent motion was labelled in the fundus of the most apical portion of the posterior insula, corresponding to the PIC+ complex of the visual-vestibular network [39–41,43,44]. The complex comprises two different foci named PIVC and PIC respectively, that can be differentiated on the selectivity to vestibular stimulation. We labelled this area as PIVC/PIC given that we used only visual and not vestibular stimulation.

For each ROI, and for both the first (coherent flow-motion versus blank) and the second stimulus (coherent versus random flow-motion), we extracted the time-courses averaged across periods of repetition, and then across subjects. We also evaluated the Signal to Noise ratio (S/N) and the phase of the response, performing an FFT on the extracted time-course before averaging. S/N was defined as the amplitude of the ratio of the fundamental harmonic and the root-mean-squared amplitude of the two frequencies closest to the fundamental [83]. The phase of the response is a good measure of the hemodynamic delay. In adults, a standard hemodynamic model corresponds to a phase value of 64 deg in our plots. The relationship between the phases of the responses across different areas and single subjects is reported in S3 Fig, both for infants and adults. To evaluate statistical significance between the adult and infant phases of the responses for each ROIs, we calculated the distances of each vector data point from the resultant of the vectorial average and its standard deviation. Then assuming a normal spread, we calculated the variation in phase associated with the predicted 2-D Normal dispersion of data around the average. Results of average, SD and *t-*tests are reported in S1 Table.

In order to consider the infants as a homogenous group, inter-subjects alignment was performed. In particular, the obtained atlas and the volumetric dataset of each subject were co-registered (by affine transformation) to one single subject (MG) with optimal quality of the anatomical image. The same transformations, one for each subject, were in turn applied to the respective functional data. One subject (DE) was discarded from this kind of analysis for poor quality of three-dimensional anatomical dataset. The 11 co-registered functional datasets were used for a multi-subjects analysis of the infants group, by using a fixed–effect (FFX) GLM-based analysis and a statistical threshold corrected for False Discovery Rate (FDR) q < 0.05 and a minimum cluster size of 81 mm^3^, corresponding to three functional voxels (Fig 5, left panel). Similarly a fixed–effect (FFX) GLM-based analysis at the same statistical threshold of q(FDR) < 0.05 and a minimum cluster size of 135 mm^3^ was performed also on adult data, after co-registration in Talairach space (Fig 5, right panel). In both infant and adult multi-subjects GLM analysis, no mask was applied. In agreement with previous work that show a great variability of the PIC+ complex location and selectivity between subjects [43], the statistical threshold of the multi-subject GLM had to be reduced to *p* < 0.05 uncorrected before activity of similar cluster-size could be identified at the expected PIVC/PIC anatomical location in infants and in adults.

Resting state series were used to study the correlation between the ROIs described above. For each subject and for each pair of ROIs, the cross correlation of the signals extracted in the resting state was calculated and the value reported in the correlation matrix. Note that given the high-pass temporal filtering the functional connectivity maps could also show a negative correlation. The striking negative correlation between V1 and MT+ in adults that is often reported also depends on the state of the subject (fixation versus eyes-closed) [56]. Note that the resting state in infants, but not in adults, was acquired during sleep. Two mean correlation matrices were obtained averaging the single-subject matrices for infants and adults, respectively. In order to achieve a value of significance for each value in the mean correlation matrices, for each group of subjects, the signals of all single subjects were normalized and concatenated, obtaining the signal of a super-subject for each ROI. The calculation of cross correlation of these signals between each pair of ROIs corresponds to the average across subjects of the correlation matrices and gives the significance of the values in terms of *p*-value. Statistical analysis (two-tailed *t*-test) was performed between correlation matrices of the two groups of subjects.

## Supporting Information

S1 Data The dataset with original data for each of the figures in the manuscript and supporting information. (XLSX)

S2 Data The dataset contains the Brain Voyager t-value images (nifti-1 format) of the statistical maps of Fig 4. (ZIP)

S3 Data The dataset contains the Brain Voyager R-value images (nifti-1 format) of the correlation maps of S2 Fig. (ZIP)

S4 Data The dataset contains the DARTEL infant brain atlas (analyse format) showed in S4 Fig. (ZIP)

S1 Fig Average time-course across subjects and individual Signal to Noise ratio for V1 seed area. A: Average time-course for V1 seed in infants (left) and adults (right) for the high contrast flow-motion versus blank stimulus of equal luminance (*p* < 0.001 in infant and *p* < 10^−10^ in adults). The bars show ±1 sem, the grey bar indicates the duration of coherent motion. B: Signal to Noise ratio polar plot for individual subjects (left column infants, right column adults). The phases of the polar plot data are similar for adults and infants, indicating that BOLD responses have a comparable hemodynamic (S1 Table). Filled symbols correspond to *p* < 0.01, open to 0.01 < *p* < 0.05 threshold. (V1: primary visual cortex; s: seconds.) Numerical data are available in S1 Data, S1 Fig.
(TIF)

S2 Fig Examples of correlation maps used as functional mask. Examples of correlation maps for the infants (CT and MG in Fig 1; FG and CG in Fig 2) used as functional mask in the General Linear Model for coherent flow versus random motion. Maps were calculated by using the cross-correlation of the V1 response to flow-motion versus blank (V1-seed) with the response of all brain voxels, using 0s temporal delays and imposing a threshold value of *p* < 0.05 (corresponding to a R value > |0.25|, as indicated in the colour bar). For each infants, the V1-seed ROI is depicted in purple. The numbers on top refer to the “ζ” coordinate of each column, the distance along the z-direction, in millimetres, from the AC point. R-L in radiological convention. (R: right; L: left; AC: anterior commissure.) Data of the statistical maps are available in S3 Data.
(TIF)

S3 Fig Comparison of the individual BOLD response phases for the various stimuli for each ROI. Top panel: Flow motion versus random motion; bottom panel: flow motion versus blank; infants on the left column, adults on the right column. Corresponding data points of ROIs in the two hemispheres (MT, V6, PIVC/PIC) were connected by a line, with the left point referring to the right hemisphere. Each colour corresponds to a single subject given by the legend below the figure. Note that phases were consistent across homologous areas for each single subject. V1-seed had a consistent phase for the coherent flow motion versus blank in adults and infants, but varied considerably for the coherent versus random flow motion in infants but not in adults. For statistical significance tests see the main text. For MT and V6 ROIs the phases of five infants are positive in response of coherent flow versus random motion, but negative in response to flow motion versus blank, suggesting that negative bold may be stimulus dependent and specific to contrast modulation. (V1s: primary visual cortex seed; MT+: temporo-occipital complex; V6: Visual area six on medial-parieto-occipital region; PIVC/PIC: posterior insular vestibular cortex/posterior insular cortex; PCu/Cu: Pre-Cuneus/Cuneus; deg: degrees.) Numerical data are available in S1 Data, S3 Fig.
(TIF)

S4 Fig Infant brain atlas. Axial sections of the brain atlas obtained by the DARTEL algorithm, using anatomical T1- weighted images of twelve infants (mean age = 7.8 ± 1.2 wk). The location along the z-direction of each axial slice is reported in millimetres from the AC point (ζ coordinate as in Table 1 main text). (R: right; L: left; AC: anterior commissure.) Data are available in S4 Data.
(TIF)

S1 Movie Example of motion stimuli. The initial segments show an example of the motion stimuli used in the infant fMRI experiment, with the first 7 s showing random motion and the subsequent 7 s the coherent flow. Note the strong vection perception elicited by the coherent flow. The segments from 15 s to 52 s show an example of the recorded eye movements of an infant during the presentation of the coherent versus random motion. Note the reflection of the stimuli on the infant’s forehead.
(WMV)

S1 Table Phase of the vectorial average across subject for each ROIs. Phase (in degrees) of the resultant vector from the vectorial average of all data of Figs 1, 3 and S1. The resultant vectors were calculated for infants (left) and adults (middle), combining left and right hemispheres. For variance estimation, we calculated the distances of each vector data point from the resultant of the vectorial average and its standard deviation. From the predicted 2-D Normal dispersion of points around the average, we calculated the corresponding variation in phase. For each ROI, the table reports mean phases of the responses to coherent versus random flow motion (random) and also the mean phases of the responses to coherent flow-motion versus blank for V1-seed. On the right, results of two-tailed *t*-tests performed between infant and adult data. Phases are in degrees. Two levels of statistical significance are shown: *p* < 0.05 (*) and *p* < 0.001 (***). (MT+: temporo-occipital complex; V6: Visual area six on medial-parieto-occipital region; PIVC: posterior insular vestibular cortex; PCu/Cu: Pre-Cuneus/Cuneus; V1: primary visual cortex; SD: standard deviation; N: number of data points; p: p-value; t: t value; DoF: degrees of freedom.)
(XLSX)

S2 Table Estimation of head movement for each subject. Evaluation of head movement for each infant (top) and adult (bottom) in the used time courses recorded during the coherent flow motion versus blank (A), the coherent versus random flow-motion (B) and during the resting-state condition (C). For A and B, half stimuli periodicity intervals of time courses with movements of translation greater than 4 mm along any directions (X, Y, Z, columns on the left) or with rotations greater than 5° respective to any axis (X, Y, Z, columns on the right) were cut and discarded; for C, single data points with above-threshold movement were discarded. The table reported the mean and standard deviation of the six rigid body parameters as well as the registered maxima value for translation and rotation. (mm: millimetres; deg: degrees; SD = standard deviation; max: maximum.)
(XLSX)

## Abbreviations

AC-PC: anterior/posterior commissure
BOLD: Blood oxygenated level dependent
deg/s: degree per seconds
DTI: Diffusion Tensor Imaging
ERP: event related potential
FDR: False Discovery Rate
FFX: fixed-effect
fMRI: functional Magnetic Resonance Imaging
GLM: General Linear Model
Hz: hertz
LCD: Liquid Crystal Display
LOd: dorsal part of the Lateral Occipital area
LOv: ventral part of the Lateral Occipital area
ms: milliseconds
MST: Medial Superior Temporal area
NIRS: Near Infrared Spectroscopy
OKN: opto-kinetic eye movements
PIC: posterior insular cortex
PIVC: posterior insular vestibular cortex
ROI: Region of Interest
S/N: Signal to Noise
V1: primary visual cortex
V3: Visual area three
V4: Visual area four
V5/MT+: temporo-occipital complex
V6: Visual area six
