# Supplementary figures and images for: BOLD Response Selective to Flow-Motion in Very Young Infants
Source: PLoS Biol. 2015 Sep 29;13(9):e1002260. doi: 10.1371/journal.pbio.1002260 (PMC4587790; doi:10.1371/journal.pbio.1002260)

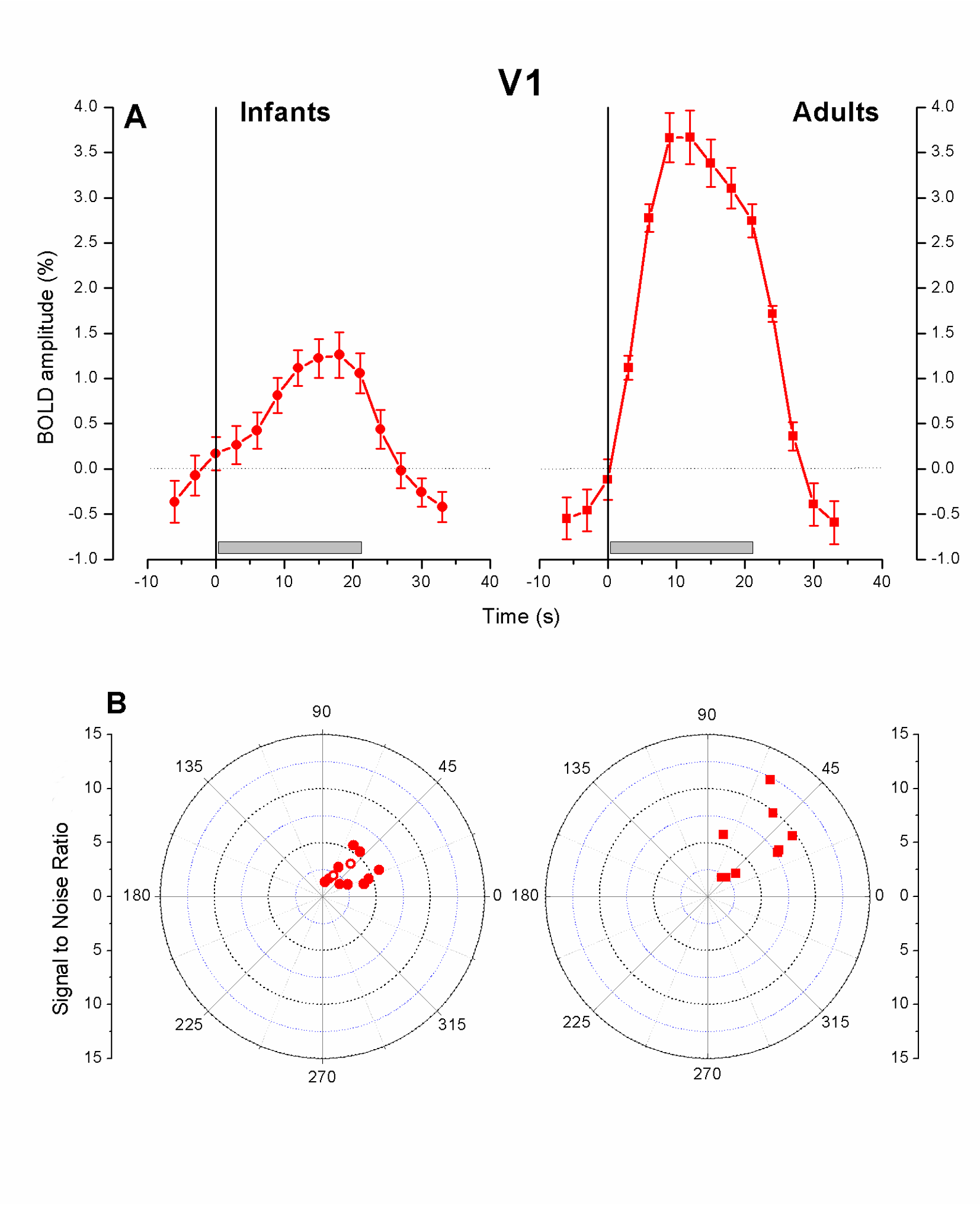

Supplement: S1 Fig — A: Average time-course for V1 seed in infants (left) and adults (right) for the high contrast flow-motion versus blank stimulus of equal luminance (p < 0.001 in infant and p < 10−10 in adults). The bars show ±1 sem, the grey bar indicates the duration of coherent motion. B: Signal to Noise ratio polar plot for individual subjects (left column infants, right column adults). The phases of the polar plot data are similar for adults and infants, indicating that BOLD responses have a comparable hemodynamic (S1 Table). Filled symbols correspond to p < 0.01, open to 0.01 < p < 0.05 threshold. (V1: primary visual cortex; s: seconds.) Numerical data are available in S1 Data, S1 Fig. (TIF) [file pbio.1002260.s005.tif]

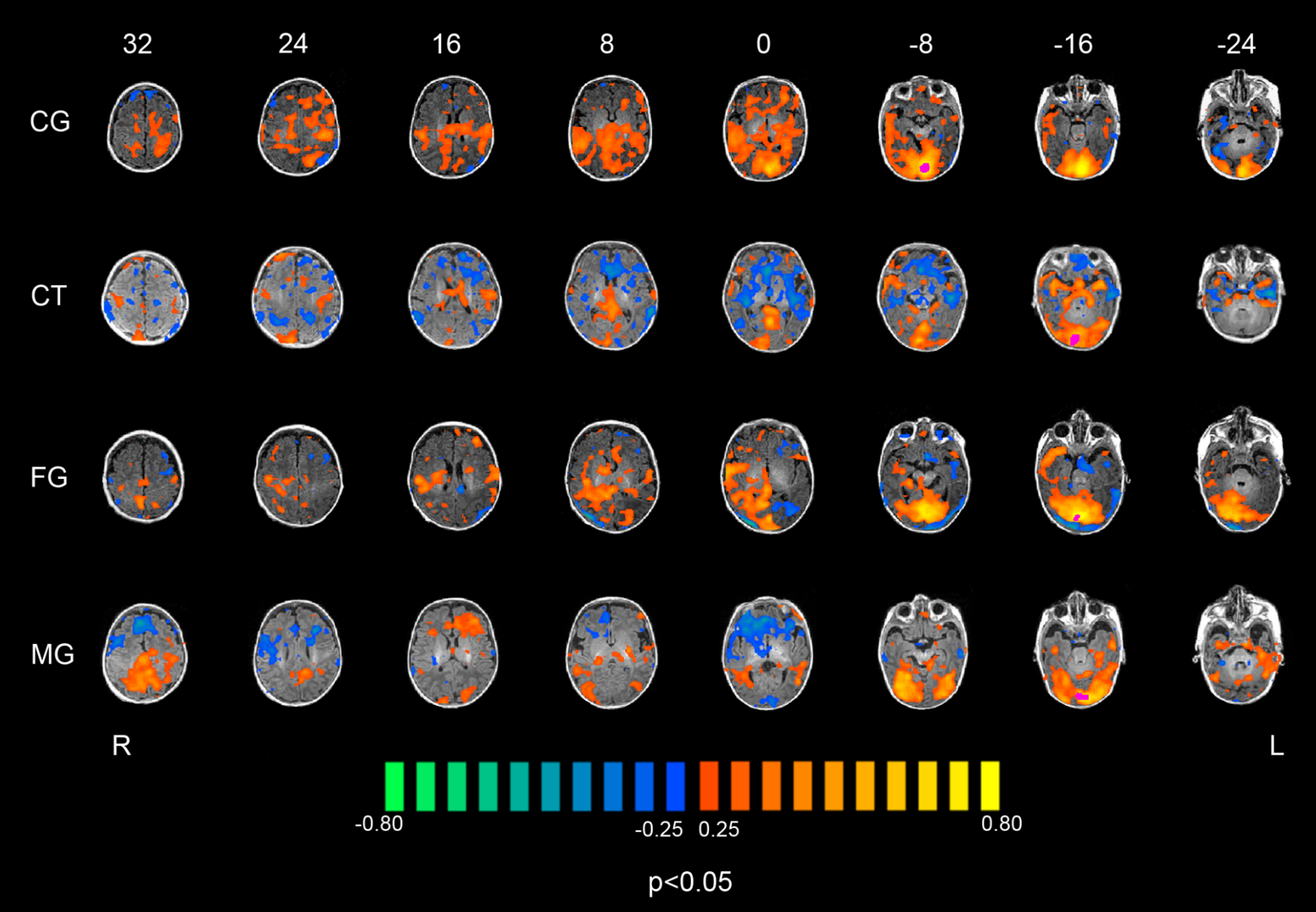

Supplement: S2 Fig — Examples of correlation maps for the infants (CT and MG in Fig 1; FG and CG in Fig 2) used as functional mask in the General Linear Model for coherent flow versus random motion. Maps were calculated by using the cross-correlation of the V1 response to flow-motion versus blank (V1-seed) with the response of all brain voxels, using 0s temporal delays and imposing a threshold value of p < 0.05 (corresponding to a R value > |0.25|, as indicated in the colour bar). For each infants, the V1-seed ROI is depicted in purple. The numbers on top refer to the “ζ” coordinate of each column, the distance along the z-direction, in millimetres, from the AC point. R-L in radiological convention. (R: right; L: left; AC: anterior commissure.) Data of the statistical maps are available in S3 Data. (TIF) [file pbio.1002260.s006.tif]

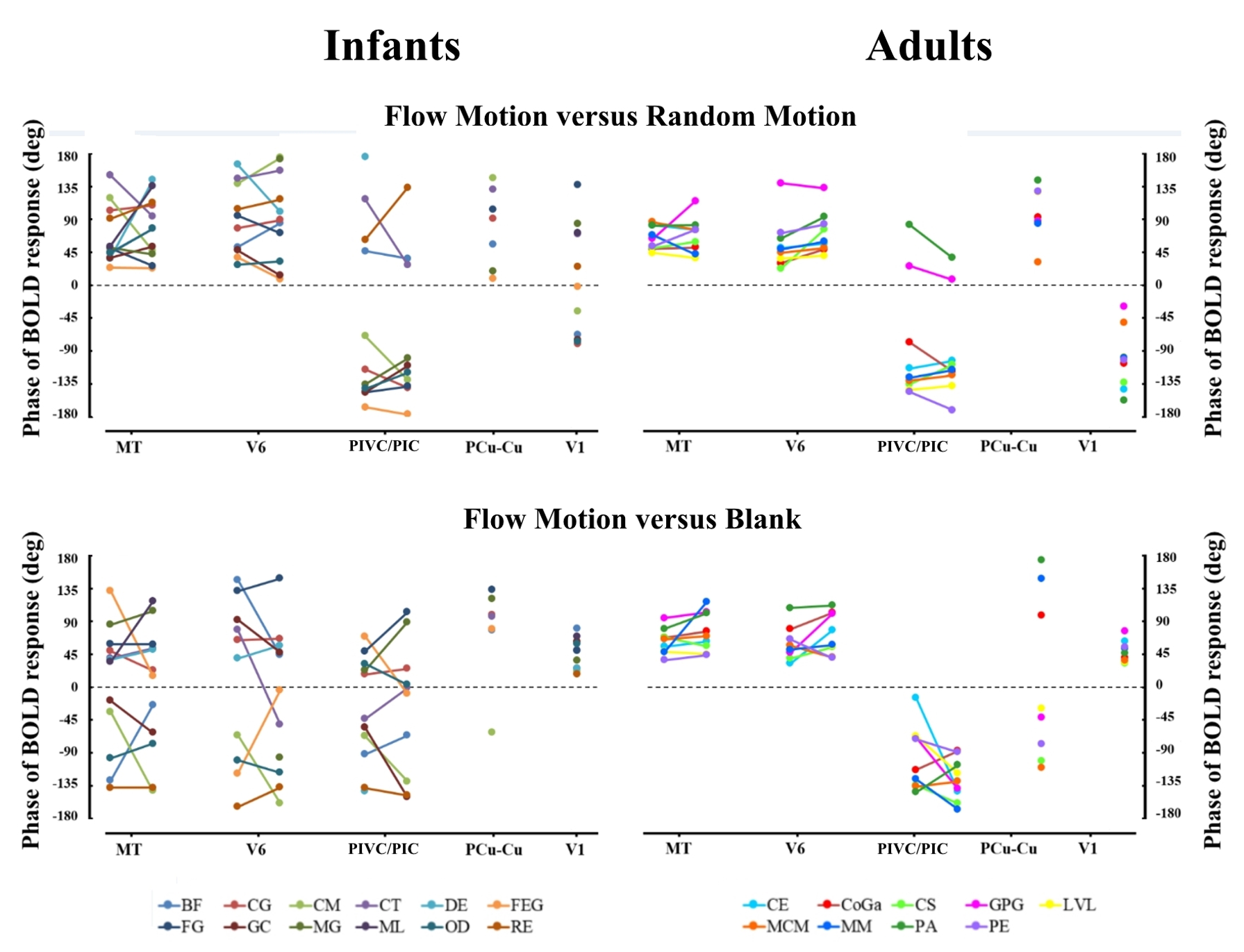

Supplement: S3 Fig — Top panel: Flow motion versus random motion; bottom panel: flow motion versus blank; infants on the left column, adults on the right column. Corresponding data points of ROIs in the two hemispheres (MT, V6, PIVC/PIC) were connected by a line, with the left point referring to the right hemisphere. Each colour corresponds to a single subject given by the legend below the figure. Note that phases were consistent across homologous areas for each single subject. V1-seed had a consistent phase for the coherent flow motion versus blank in adults and infants, but varied considerably for the coherent versus random flow motion in infants but not in adults. For statistical significance tests see the main text. For MT and V6 ROIs the phases of five infants are positive in response of coherent flow versus random motion, but negative in response to flow motion versus blank, suggesting that negative bold may be stimulus dependent and specific to contrast modulation. (V1s: primary visual cortex seed; MT+: temporo-occipital complex; V6: Visual area six on medial-parieto-occipital region; PIVC/PIC: posterior insular vestibular cortex/posterior insular cortex; PCu/Cu: Pre-Cuneus/Cuneus; deg: degrees.) Numerical data are available in S1 Data, S3 Fig. (TIF) [file pbio.1002260.s007.tif]

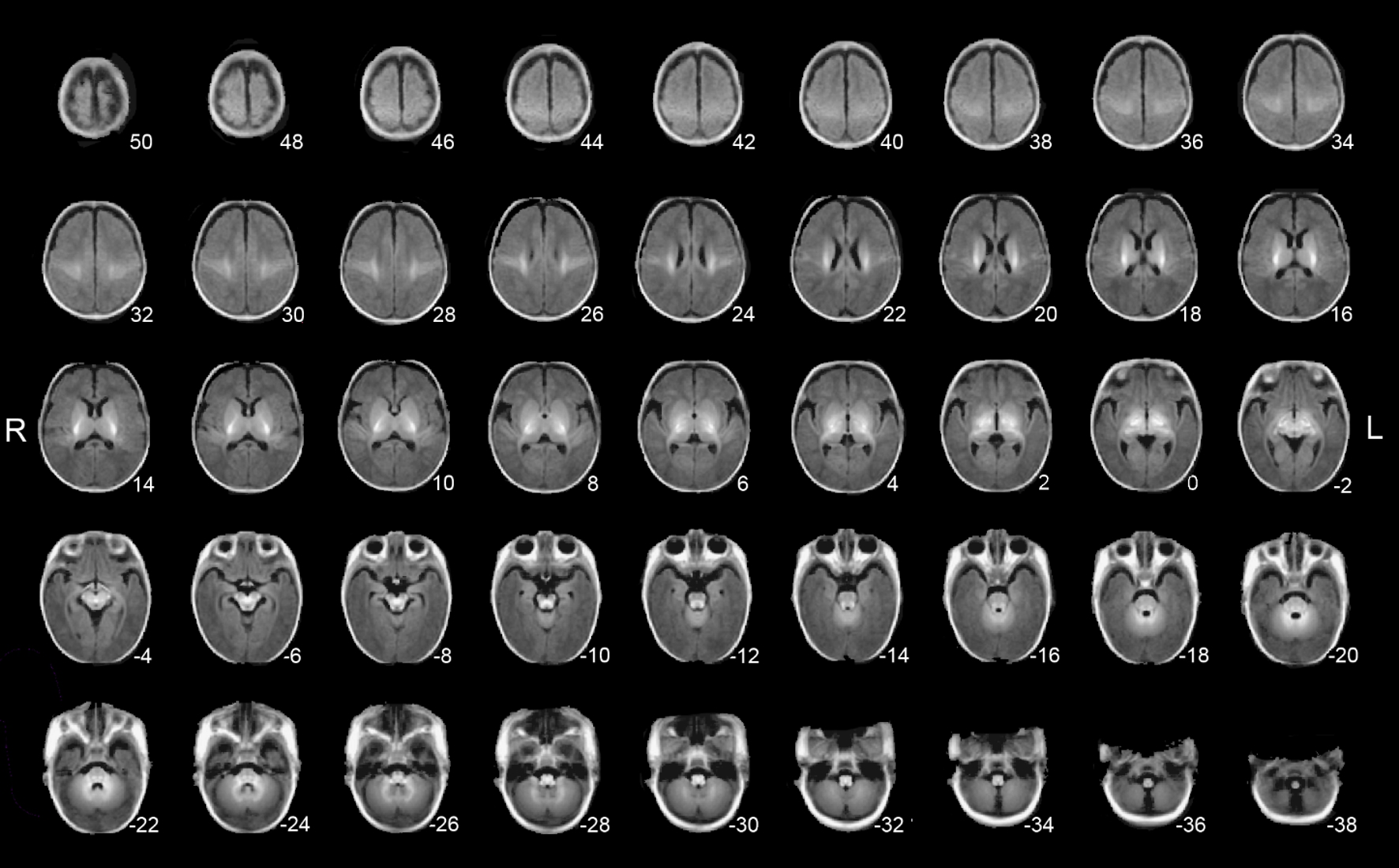

Supplement: S4 Fig — Axial sections of the brain atlas obtained by the DARTEL algorithm, using anatomical T1- weighted images of twelve infants (mean age = 7.8 ± 1.2 wk). The location along the z-direction of each axial slice is reported in millimetres from the AC point (ζ coordinate as in Table 1 main text). (R: right; L: left; AC: anterior commissure.) Data are available in S4 Data. (TIF) [file pbio.1002260.s008.tif]
